# Food allergens in mattress dust in Norwegian homes: a significant source of allergen exposure?

**DOI:** 10.1186/2045-7022-3-S3-O3

**Published:** 2013-07-25

**Authors:** RJ Bertelsen, CK Faeste, B Granum, E Egaas, SJ London, K-H Carlsen, KL Carlsen, M Lovik

**Affiliations:** 1 Dept. Food, Water and Cosmetics, Norwegian Institute of Public Health, Oslo, Norway; 2 Dept. Chemistry and Toxicology, Norwegian Veterinary Institute, Oslo, Norway; 3 National Institute of Environmental Health Sciences, National Institutes of Health, Dept. of Health and Human Services, Research Triangle Park, Durham, NC, USA; 4 Dept. Pediatrics, Oslo University Hospital, Oslo, Norway

## Background

Sensitization to food allergens is supposed mostly to be caused by ingestion of the allergen. However, sensitization may also be initiated in the respiratory tract or through the skin. Little is known about sources and prevalence of food allergens in environmental samples. We aimed to describe the presence of food allergens in mattress dust from the homes of 13 year old Norwegian adolescents in relation to home characteristics.

## Methods

Food allergens from egg, peanut, fish, and milk were measured by dot-blot analysis in mattress dust from 143 homes of 13-year olds in Oslo, Norway. The results of the dot-blot analyses were semi-quantifiable and dichotomized into *no detection* and *confirmed detection*. Associations between food allergen detection and home characteristics (collected by study investigators and by parental questionnaires) were assessed by chi-square tests and by multivariate logistic regression models.

## Results

Fish allergen was found in 46%, peanut in 41%, milk in 39%, and egg allergen in 22% of the mattresses, and only three dust samples contained none of the four food allergens. Milk allergen was more likely to be found in mattresses from beds that are usually made (covered) during the day (53%) than in beds that are not covered during the day (31%), p=0.01. All four food allergens were more frequently detected in small dwellings (<100m^2^) compared to larger dwellings (≥130 m^2^). Milk, peanut, and egg allergens were more frequently detected in homes with kitchen and bedroom on the same floor as compared to homes with kitchen and bedroom on separate floors; the adjusted odds ratios were 2.6 (95% confidence interval (CI): 1.2, 5.7) for milk allergen, 2.6 (95% CI: 1.3, 5.3) for peanut allergen, and 3.1 (95% CI: 1.3, 7.2) for egg allergen.

## Conclusion

Food allergens occurred frequently in beds in Norwegian homes, with dwelling size and proximity of kitchen and bedroom as the most important determinants. To our best knowledge, our study is the first to demonstrate allergens from fish, peanut, egg, and milk to be frequently present in mattresses dust. Because children spend so much time in the bedroom our findings suggest that food allergens in mattress dust may be an important route of exposure.

## Disclosure of interest

None declared.

